# Gut Microbiota Alterations and Reproductive Tract Dysbiosis in Endometriosis: A Systematic Review

**DOI:** 10.3390/medicina62020351

**Published:** 2026-02-10

**Authors:** Beatrice Crestani, Stefano Uccella, Matteo Pavone, Fabio Barra, Silvia Baggio, Marcello Ceccaroni, Filippo Alberto Ferrari

**Affiliations:** 1 Department of Obstetrics and Gynecology, Azienda Ospedaliera Universitaria Integrata Verona, University of Verona, 37126 Verona, Italy; 2 UOC Ginecologia Oncologica, Dipartimento di Scienze per la Salute Della Donna e del Bambino e di Sanità Pubblica, Fondazione Policlinico Universitario A. Gemelli, IRCCS, 00168 Rome, Italy; 3 IHU Strasbourg, Institute of Image-Guided Surgery, 67000 Strasbourg, France; 4 Research Institute Against Digestive Cancer (IRCAD), 67000 Strasbourg, France; 5 Department of Obstetrics and Gynecology, Gynecologic Oncology and Minimally Invasive Pelvic Surgery, International School of Surgical Anatomy, IRCCS “Sacro Cuore” Hospital, Negrar di Valpolicella, 37024 Verona, Italy; 6 Department of Health Sciences (DISSAL), University of Genoa, 16132 Genoa, Italy

**Keywords:** endometriosis, gut microbiota, microbial dysbiosis, estrobolome, β-glucuronidase activity, endometriosis-associated infertility

## Abstract

*Background and Objectives*: Endometriosis is a chronic, estrogen-dependent inflammatory disease with multifactorial pathogenesis. Increasing evidence suggests that alterations in the gut and reproductive tract microbiota may contribute to disease development, progression, and associated symptoms through immune, hormonal, and metabolic mechanisms. This systematic review aimed to synthesize current human evidence on microbiota composition and function in women with endometriosis. *Materials and Methods*: A systematic literature search was conducted according to PRISMA 2020 guidelines across PubMed, Embase, Web of Science, Scopus, and the Cochrane Library. Observational human studies published in English between January 2015 and September 2025 evaluating gut, vaginal, cervical, endometrial, or peritoneal microbiota in women with endometriosis were included. Two reviewers independently screened studies, extracted data, and performed a qualitative synthesis due to methodological heterogeneity. *Results*: Nineteen studies were included, encompassing gut and reproductive tract samples analyzed primarily by 16S rRNA sequencing. Across cohorts, endometriosis was consistently associated with microbial dysbiosis characterized by enrichment of Proteobacteria and Firmicutes and depletion of Bacteroidetes, Lactobacillus, and Bifidobacterium. Increased abundance of opportunistic taxa, particularly *Escherichia coli*, *Streptococcus*, and *Klebsiella*, was frequently reported. Functionally, dysbiosis was linked to increased β glucuronidase activity, enhanced estrogen enterohepatic recirculation, reduced short-chain fatty acid production, and activation of pro-inflammatory immune pathways. Several studies reported correlations between microbial profiles, disease stage, pelvic pain, and infertility. *Conclusions*: Current evidence supports a reproducible association between gut microbiota dysbiosis and endometriosis. Altered microbial composition and function may contribute to chronic inflammation, hormonal imbalance, and disease persistence. Longitudinal and multi-omic studies are needed to clarify causality and to evaluate microbiota-based diagnostic and therapeutic strategies.

## 1. Introduction

Endometriosis is a chronic, estrogen-dependent gynecological disorder characterized by the presence of an endometrium-like epithelium and/or stroma outside the endometrium and myometrium, usually with an associated inflammatory process [1,2,3,4]. Affecting approximately 10% of women of reproductive age [5,6], it is associated with significant morbidity, including chronic pelvic pain, dysmenorrhea, dyspareunia, and infertility [7,8,9,10,11,12]. While the precise pathogenesis of endometriosis remains unclear, multiple theories have been proposed, including retrograde menstruation, coelomic metaplasia, immune dysfunction, and environmental factors [13,14,15,16,17].

In recent years, increasing evidence has suggested that the human microbiome, particularly the gut and reproductive tract microbiota, may play a pivotal role in the development and progression of endometriotic implants [18,19]. Dysbiosis, or microbial imbalance, has been implicated in systemic inflammation, immune dysregulation, and alterations in estrogen metabolism, all of which are integral to endometriosis pathophysiology [20,21,22].

An overrepresentation of Gram-negative bacteria such as *Escherichia coli*, *Pseudomonas*, and *Prevotella* has been reported in the gut, cervical mucus, and peritoneal fluid of affected women, consistent with findings from both human and murine models [23,24,25]. These species are known to express β-glucuronidase, an enzyme that deconjugates estrogens in the gut, thereby increasing circulating active estrogen levels [26,27]. Moreover, an elevated *Firmicutes*/*Bacteroidetes* ratio and reduced microbial diversity have been consistently reported across gut, vaginal, cervical, and peritoneal samples from affected individuals [28,29,30,31].

Despite growing interest, there remains a lack of studies that synthesize current evidence across anatomical sites and microbial taxa. Additionally, the role of the estrobolome, the collection of enteric bacterial genes involved in estrogen metabolism, remains underexplored in the context of endometriosis [22]. The aim of this systematic review is to synthesize the existing literature on the composition and diversity of the gut and reproductive tract microbiota in women with endometriosis.

## 2. Materials and Methods

This systematic review was conducted in accordance with the Preferred Reporting Items for Systematic Reviews and Meta-Analyses (PRISMA) 2020 guidelines. The review protocol was not registered in any public database.

### 2.1. Eligibility Criteria

Studies were eligible for inclusion if they investigated the composition, diversity, or function of the gut or reproductive tract microbiota, including vaginal, cervical, endometrial, or peritoneal samples, in women with a clinical or histological diagnosis of endometriosis. Eligible study designs included observational human studies, including case–control, cross-sectional, or cohort designs, with or without a comparison group of healthy controls. Only original articles published in English from January 2015 to September 2025 were considered. Studies were excluded if they were review articles, meta-analyses, case reports, conference abstracts, editorials, guidelines, consensus statements, or if they involved animal or in vitro models.

### 2.2. Information Sources and Search Strategy

A comprehensive literature search was carried out in PubMed, Embase, Scopus, Web of Science, and Cochrane Library databases, from inception to 15 September 2025. The search strategy included both MeSH terms and keywords related to endometriosis and microbiota, such as: “endometriosis”, “gut microbiota”, “intestinal microbiome”, “vaginal microbiota”, “endometrial microbiome”, “microbiota-gut-brain axis”, “estrobolome”, and “microbiome dysbiosis”. The search was supplemented by manual screening of reference lists from relevant articles. The full search strings used for each database are available upon request. No automation tools or AI-based filters were applied during the search process.

### 2.3. Study Selection

**Figure 1:**
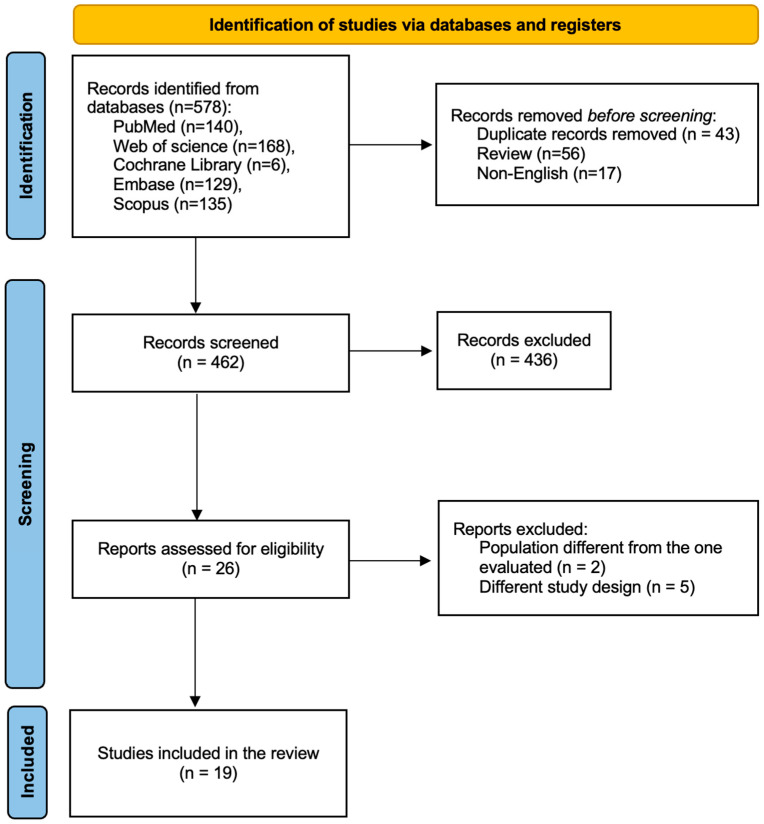
PRISMA flow diagram for study selection.

All retrieved references were imported into citation management software, and duplicates were removed. Two reviewers (B.C. and F.A.F.) independently screened titles and abstracts for potential eligibility. Full-text articles were then assessed in detail against the inclusion and exclusion criteria. Discrepancies between reviewers were resolved by consensus or through discussion with a third reviewer (S.U.). The overall selection process is illustrated in the PRISMA 2020 flow diagram (Figure 1), including the number of records identified, screened, assessed for eligibility, and ultimately included in the review.

### 2.4. Data Collection Process

Data extraction was performed independently by two reviewers using a standardized extraction form. Extracted data included: first author and year of publication, study location, study design and sample size, microbiota sampling site (e.g., fecal, vaginal, endometrial), methodology used for microbial analysis (e.g., 16S rRNA sequencing, targeted region, sequencing platform), and key findings related to microbial composition, diversity, and functional profiles. The extracted data were cross-verified by a second reviewer to ensure consistency and accuracy. No automated tools were employed during the data collection phase.

### 2.5. Data Items

The primary outcomes assessed in this review were: (i) alterations in microbial composition and diversity metrics (e.g., alpha and beta diversity), (ii) identification of taxa associated with endometriosis presence or severity, and (iii) functional aspects of the microbiome, such as β-glucuronidase activity, estrogen metabolism, or production of short-chain fatty acids. Secondary outcomes included microbiota associations with clinical features such as pelvic pain, infertility, and stage of endometriosis.

### 2.6. Risk of Bias Assessment

The methodological quality and risk of bias of the included studies were evaluated using the Newcastle–Ottawa Scale (NOS), a validated instrument designed to assess non-randomized studies included in systematic reviews. The NOS examines three main domains: the selection of study participants, the comparability of study groups, and the ascertainment of exposure or outcomes, depending on study design. Each study was rated on a scale with a maximum score of nine points. Studies receiving a score of six or above were considered to be of moderate to high methodological quality. The assessment was conducted independently by two reviewers, with any disagreements being resolved through discussion and consensus. Particular attention was given to the adequacy of participant selection, clarity of inclusion and exclusion criteria, control for potential confounders, and the robustness of outcome measurement. A summary table of NOS ratings for all included studies is provided in the Section 3.

### 2.7. Synthesis Methods

**Figure 2:**
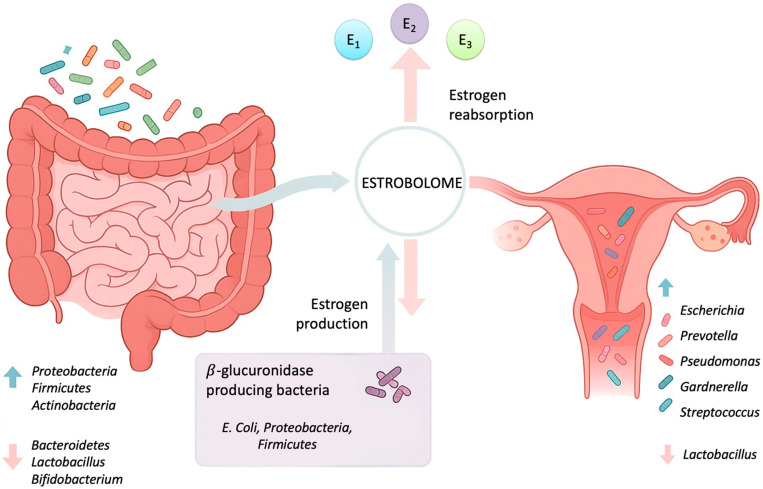
Conceptual representation of the estrobolome and its interaction with gut and reproductive tract microbiota. The left panel illustrates gut microbial shifts associated with dysbiosis, including increased abundance of Proteobacteria, Firmicutes, and Actinobacteria and decreased levels of Bacteroidetes, Lactobacillus, and Bifidobacterium. These alterations enhance β-glucuronidase activity and promote deconjugation of estrogen metabolites, facilitating their reabsorption. The central schematic highlights the estrobolome as the functional microbial network regulating estrogen production (E1, E2, E3) and enterohepatic recycling. The right panel depicts reproductive tract dysbiosis, characterized by enrichment of Escherichia, Prevotella, Pseudomonas, Gardnerella, and Streptococcus, with reduced Lactobacillus representation.

Given the substantial heterogeneity in study designs, microbial targets, sequencing methods, and clinical outcomes, a quantitative meta-analysis was not feasible. Instead, findings were synthesized, grouping studies based on the anatomical site of microbiota sampling (e.g., gut vs. reproductive tract), the taxonomic or functional shifts identified, and associated clinical phenotypes. No subgroup analyses, sensitivity analyses, or meta-regression were performed. Key characteristics of the included studies are summarized in Table 1, and a visual summary of microbial findings is provided in Figure 2.

## 3. Results

We identified 578 manuscripts with the initial systematic literature search. After duplicate removal and title and abstract screening, we reviewed the full text of 25 manuscripts. Finally, we included 19 papers in the final synthesis (Table 1). Nine case–control studies were included, along with nine cohort studies and one cross-sectional study. Due to significant heterogeneity between studies, quantitative data synthesis was not possible. We performed a qualitative synthesis, organized in thematic sections, to summarize the available evidence. Study quality was generally moderate to high, with many incorporating external validation and clinical comparisons. Complete risk-of-bias assessment is reported in Table S1.

### 3.1. Overview of Gut Microbiota and Its Relevance to Endometriosis

The human gut microbiota is a complex and dynamic ecosystem composed of trillions of microorganisms that regulate nutrient metabolism, immune homeostasis, and endocrine balance [18,19]. It is dominated by *Firmicutes* and *Bacteroidetes*, with smaller contributions from *Actinobacteria*, *Proteobacteria*, and *Fusobacteria* [46]. Alterations in this microbial network, referred to as dysbiosis, are associated with chronic inflammatory and metabolic disorders, including obesity, inflammatory bowel disease, and endocrine dysfunctions [18]. Given the crosstalk between microbial, hormonal, and immune pathways, the gut microbiome has emerged as a critical component in the multifactorial pathogenesis of endometriosis [2,20,22]. Baușic et al. demonstrated that women with endometriosis exhibit reduced diversity and enrichment of pro-inflammatory taxa such as *Escherichia coli*, highlighting the systemic inflammatory link between gut imbalance and disease progression [45]. Similarly, Valdés-Bango et al. observed overlapping microbial alterations between gut and reproductive sites in adenomyosis [41].

### 3.2. Dysbiosis of the Gut Microbiota in Endometriosis

Multiple studies have consistently documented the presence of microbial dysbiosis in women with endometriosis, revealing a reproducible shift in gut microbial composition marked by an increased relative abundance of Proteobacteria and Firmicutes together with a reduction in Bacteroidetes and Actinobacteria [46,47]. This altered taxonomic profile reflects a disruption of microbial homeostasis that has been observed across different cohorts and analytical approaches. Within this dysbiotic framework, *Escherichia coli* has been repeatedly identified as significantly enriched, most notably in the investigations conducted by Akiyama et al. and Khan et al. [32,48]. The overrepresentation of *E. coli* supports its proposed contribution to disease pathophysiology through lipopolysaccharide-mediated immune activation and the amplification of inflammatory signaling pathways [19,49]. In addition to *E. coli*, other pathogenic and opportunistic taxa, including *Streptococcus*, *Klebsiella*, and *Enterococcus*, are frequently reported to be increased in abundance in endometriosis-associated microbiomes [18,50]. Conversely, several beneficial commensal microorganisms are consistently depleted. These include *Lactobacillus* and *Bifidobacterium*, which are known to exert protective immunomodulatory effects, as well as *Faecalibacterium* and short-chain fatty acid-producing *Clostridia*, which play a critical role in maintaining intestinal metabolic balance and epithelial health [18,50]. The loss of these beneficial taxa, combined with the expansion of pro-inflammatory and opportunistic bacteria, is thought to promote a microbiome characterized by reduced metabolic capacity and heightened inflammatory potential. Such alterations may compromise epithelial barrier integrity, increase intestinal permeability, and disrupt immune regulation, thereby contributing to systemic inflammation and disease progression in endometriosis [22,28]. Alpha-diversity findings remain inconsistent, likely due to methodological variation [21,50], whereas beta-diversity analyses robustly differentiate endometriosis from control microbiota [19,46]. Functionally, increased β-glucuronidase activity and reduced short-chain fatty acid (SCFA) production suggest impaired mucosal defense and enhanced estrogen recycling [26,27]. Notably, microbial profiles appear to evolve with disease progression. Ata et al. found more pronounced dysbiosis in stage III–IV disease according to revised American Society for Reproductive Medicine (rASRM) classification [28], while Chang et al. and Perrotta et al. identified distinct microbial shifts across clinical stages, suggesting a progressive remodeling of microbial communities alongside disease severity [34,37]. Chang et al. found that advanced disease is marked by reduced microbial diversity and dominance of *Lactobacillus jensenii*, a species potentially adapted to inflammatory, estrogen-rich conditions. This overrepresentation reflects dysbiosis, not a healthy state, as *L. jensenii* may impair mucosal integrity and sustain low-grade inflammation. Perrotta et al., conversely, observed a loss of Lactobacillus-dominated communities and a rise in anaerobes such as *Anaerococcus* with increasing disease severity. Consequently, the repeated identification of specific bacterial taxa across independent cohorts supports the feasibility of microbiota-based diagnostic markers. Elevated *E. coli* and *Streptococcus*, along with reduced *Lactobacillus* and *Bifidobacterium*, have been proposed as potential microbial signatures of endometriosis [18,46]. Supporting this, Hicks et al. identified distinct oral, vaginal, and stool microbial signatures in endometriosis, particularly enrichment of Fusobacterium in moderate-to-severe disease, highlighting the diagnostic potential of multisite microbial profiling [39]. Malvezzi et al. further detected *Flavobacterium*, *Pseudomonas*, and *Bacillus* in peritoneal fluid, linking microbial colonization directly with the pelvic inflammatory milieu [43]. Metabolomic profiling adds a functional dimension, showing altered levels of bile acids, tryptophan metabolites, and SCFAs that distinguish affected women from controls [19,49].

### 3.3. The Estrobolome: Microbial Control of Estrogen Metabolism

The *estrobolome*, the subset of gut microbial genes capable of metabolizing estrogens, provides a mechanistic link between the gut microbiota and endometriosis [26,27] (Figure 2). Alterations in this microbial network can disrupt systemic estrogen homeostasis and trigger inflammatory cascades central to endometriosis pathogenesis [51]. Bacteria such as *E. coli* expressing β-glucuronidase enzymes can deconjugate estrogens in the gut, allowing their enterohepatic recirculation and increasing systemic estrogen availability [19,46]. This enzymatic activity, particularly associated with taxa from the *Firmicutes* and *Bacteroidetes phyla*, promotes excessive estrogen reactivation that sustains ectopic endometrial growth and local immune dysregulation. Nannini et al. report that dysbiosis may be associated with increased β-glucuronidase activity, which in turn has been linked to enhanced estrogenic signaling via ERβ pathways, disrupted epithelial barrier integrity, and elevated systemic inflammation [51]. These data support a causal role for microbial estrogen metabolism in maintaining endometriotic lesions [8,32,48]. Pai et al. confirmed that women with endometriosis exhibit increased fecal β-glucuronidase activity and elevated estrogen metabolites [38], and similarly, Le et al. linked fluctuations in urinary estrogens and microbial dynamics, suggesting that gut microbiota composition directly influences estrogen bioavailability [36].

### 3.4. Gut Microbiota, Endometriosis, and Infertility

Approximately 30% of women with endometriosis experience infertility, whereas up to 50% of infertile women may have a diagnosis of endometriosis [1,6,52,53]. Gut dysbiosis may intensify mechanisms of endocrine and immune disturbances through systemic inflammatory signaling and hormonal imbalance [18,19]. Increased *Proteobacteria* abundance enhances LPS-mediated activation of Toll-like receptor 4 (TLR4)–NF-κB pathways, resulting in elevated cytokines such as interleukin (IL)-6 and Tumor Necrosis Factor (TNF)-α, which impair folliculogenesis and endometrial receptivity [2,46]. Meanwhile, depletion of SCFA-producing taxa such as *Faecalibacterium prausnitzii* compromises mucosal integrity, facilitating inflammation that disrupts implantation [19,50]. The estrobolome further contributes via microbial β-glucuronidase activity that promotes estrogen recirculation, sustaining lesion growth and perturbing ovulatory and luteal functions [26,27,48]. Reduced *Lactobacillus* dominance in the genital tract, likely linked to upstream gut imbalance, has also been associated with poor implantation outcomes [24,30]. Collectively, gut dysbiosis appears to exacerbate endometriosis-related infertility through converging inflammatory and hormonal mechanisms. Microbiota modulation through probiotics, prebiotics, or dietary intervention remains a promising but experimental strategy [7,10]. A personalized nutritional and microbiome-targeted approach aimed at modulating low-grade chronic inflammation has gained increasing attention [54]. In this context, supplementation with *Lactobacillus crispatus* has shown evidence of beneficial effects in infertile patients presenting with dysbiosis [55]. Wang et al. demonstrated that elevated IL-6, IL-10, IL-13, and TNF-α in the peritoneal fluid of infertile women with endometriosis correlated with an overrepresentation of Proteobacteria and Firmicutes, directly linking immune activation to microbial composition [33]. Jimenez et al. further showed that vaginal and rectal dysbiosis contribute to genital inflammation in chronic pelvic pain, emphasizing how gut–genital microbial crosstalk sustains reproductive dysfunction [40].

### 3.5. Gut Microbiota and Immune Modulation

The gut microbiota plays a central role in shaping immune homeostasis, and its disruption contributes to the chronic inflammatory profile observed in endometriosis [2,22]. Dysbiosis, particularly the overgrowth of Gram-negative bacteria such as *E. coli* and *Klebsiella*, increases exposure to LPS, which activates TLR4–NF-κB signaling and drives secretion of pro-inflammatory cytokines including IL-6, IL-1β, and TNF-α [19,46,49]. These cytokines promote lesion growth, angiogenesis, and local immune infiltration, sustaining the inflammatory cycle typical of endometriosis. At the same time, depletion of immunoregulatory taxa such as *Lactobacillus*, *Bifidobacterium*, and *Faecalibacterium prausnitzii* reduces production of SCFAs, which are crucial for maintaining intestinal barrier integrity and regulatory T-cell (Treg) function [19,50]. Loss of SCFA-mediated immune tolerance favors a Th17-dominant environment that amplifies inflammation [2]. This immune imbalance may also extend to the peritoneal cavity, where microbial products alter macrophage polarization and perpetuate a chronic, low-grade inflammatory state [20,46]. Toffoli et al. identified an association between low mannose-binding lectin pathway functionality and dysbiosis in endometriosis, suggesting that impaired complement activation may facilitate bacterial persistence and inflammation [44]. Do et al. also reported that hormonal factors and prior surgical interventions influence gut microbial stability, potentially modulating systemic immune responses [42]. Shan et al. confirmed that lower microbial diversity and a higher Firmicutes/Bacteroidetes ratio correlate with increased estradiol and IL-8 levels, strengthening the connection between dysbiosis, hormonal imbalance, and inflammatory activity [35].

## 4. Discussion

This systematic review collects emerging evidence suggesting a potential link between gut microbiota dysbiosis and the pathophysiology and progression of endometriosis. Among various methods for bacterial identification, 16S rRNA sequencing is a widely used technique that exploits conserved gene regions for universal primer binding and hypervariable regions for species-level classification [56]. In addition to its taxonomic utility, 16S rRNA sequencing offers practical advantages in the context of clinical application; it is increasingly cost-efficient, relatively rapid, and accessible, making it a promising tool for potential integration into routine microbiota-based diagnostic strategies. While several studies report microbial alterations associated with endometriosis severity, it is essential to interpret these findings cautiously due to their observational nature. Consistent patterns, such as depletion of protective taxa and expansion of potentially pro-inflammatory species, have been noted in multiple cohorts [28,34,37]. Yuanyue et al. and Colonetti et al. observed significant differences in microbial α- and β-diversity between women with and without endometriosis, suggesting that gut microbiota composition may be altered in affected individuals [50,57]. Nonetheless, these differences do not confirm a definitive or disease-specific microbial signature, particularly given the heterogeneity in sample types (e.g., fecal, vaginal, endometrial), sequencing platforms, population characteristics, and disease phenotypes. This variability may open a window for further reflection on the potential site-specific associations between distinct microbial communities and the heterogeneous clinical spectrum of endometriosis. Such considerations highlight the need for future studies to adopt stratified approaches based on well-defined clinical subgroups, such as disease stage, lesion localization, or symptom profiles, to better elucidate microbiota–disease relationships. A key element is the central role of the estrobolome, the microbial gene network capable of metabolizing estrogens. Dysbiosis-associated upregulation of β-glucuronidase activity, as shown by Pai et al. and Le et al., may enhance enterohepatic estrogen recirculation, a mechanism that could hypothetically reinforce the hyperestrogenic state associated with endometriosis [36,38,51]. The findings of Yuanyue et al. similarly support this axis, emphasizing that disruption of Firmicutes/Bacteroidetes balance correlates with altered glucuronidase activity and estrogen metabolism [57]. While this emerging model provides a valuable theoretical framework, causal relationships between dysbiosis, estrobolome activity, and endometriosis pathophysiology remain to be empirically confirmed. Unlike prior reviews, the present work integrates data from metagenomic and metabolomic analyses to explore not only compositional but also functional aspects of microbial dysregulation. This helps to generate plausible biological hypotheses regarding the involvement of microbial endocrine–immune crosstalk in disease mechanisms. Interventions targeting the microbiome, particularly the restoration of Lactobacillus-dominated communities through *Lactobacillus crispatus* supplementation, have shown promise in modulating estrogen metabolism and reducing inflammatory burden [54]. Moreover, supplementation with natural anti-inflammatory and antioxidant compounds (e.g., curcuma longa, coenzyme Q10, zinc), along with multi-strain probiotics like *Bifidobacterium* and *Lactobacillus*, may support gut microbiota balance and improve fertility and pregnancy outcomes [20,58,59]. However, current data remain preliminary and constrained by small cohorts, heterogeneity in sequencing approaches, and limited longitudinal evidence. The current evidence supports the hypothesis that dysbiosis may be associated with altered estrogen metabolism and systemic inflammation in women with endometriosis. Yet no consistent microbiological or estrobolomic “signature” has been established. Future research should aim to validate these pathways in longitudinal, multi-omic studies and assess targeted microbiome-based therapies as adjunctive strategies in endometriosis management.

### Limitations of the Study

Nonetheless, this study has several limitations. First, the majority of included studies are observational, predominantly cross-sectional in design, and methodologically heterogeneous. Potential biases may arise from methodological variability and uncontrolled confounding factors. For example, many studies do not distinguish between superficial and deep endometriosis, limiting the precision of the associations reported. Second, the inclusion of studies analyzing microbiota from various anatomical sites beyond fecal samples, such as vaginal, cervical, endometrial, and peritoneal samples, introduces a considerable degree of heterogeneity. While this broad approach offers a more comprehensive view of microbiota–endometriosis interactions, it also limits the ability to draw focused conclusions specifically regarding the gut microbiota. Consequently, the identification of a reproducible, site-specific microbiota profile, particularly for the gut, remains challenging. Third, while several studies explore functional mechanisms such as β-glucuronidase activity, the evidence is insufficient to support any definitive causal link between microbial shifts and disease onset or progression. Interpretations should therefore remain hypothesis-generating rather than confirmatory. Finally, the review protocol was not registered in any publicly accessible database, such as PROSPERO. Although this does not compromise the validity of the results, it constitutes a methodological limitation that warrants acknowledgment.

## 5. Conclusions

In conclusion, current evidence suggests that the gut microbiota may play a dual role in endometriosis, potentially reflecting underlying disease processes and possibly influencing their progression. Observed alterations in microbial composition have been associated with changes in estrogen metabolism, immune modulation, and metabolic pathways, which may contribute to an environment favoring chronic inflammation and sustained lesion activity. These interconnected mechanisms support the hypothesis that dysbiosis could accompany and reinforce endometriosis persistence and symptom severity. Although considerable advances have been made in defining the microbial signatures associated with the disease, a definitive understanding of causality and microbial function remains lacking. Future research that combines mechanistic investigations with well-designed clinical studies and functional microbiome analyses will be essential to clarify these associations and to assess whether targeting the gut microbiota represents a viable adjunctive approach for diagnosis or treatment in patients with endometriosis.

## Figures and Tables

**Table 1:** Characteristics of the included studies.

Authors (Year)	Country	Study Design	Sample Size (Endometriosis vs. Control)	Diagnostic Method	Sample Type	Sequencing Method	Main Findings in Endometriosis Patients
Khan et al. (2016) [32]	Japan	Case–control	32 vs. 32	Laparoscopy and histological confirmation	Endometrial swabs, cystic fluid	16S rRNA metagenomic sequencing (V4–V5 regions, Illumina MiSeq)	Detected intrauterine microbial colonization Reduced *Lactobacillaceae* Increased *Streptococcaceae*, *Staphylococcaceae*, and *Enterobacteriaceae* after GnRHa treatment
Wang et al. (2018) [33]	China	Case–control	55 vs. 30	Laparoscopy for diagnosis and infertility evaluation	Peritoneal fluid	Ion Torrent PGM 16S sequencing; ELISA	*Proteobacteria* and *Firmicutes* were predominant Increased IL-6, IL-10, IL-13, and TNF-α correlated with infertility and pro-inflammatory microbiota
Ata et al. (2019) [28]	Turkey	Cohort	14 vs. 14	Laparoscopic diagnosis (r-ASRM)	Stool, vaginal and cervical swabs	16S rRNA sequencing (V3–V4 regions)	Altered gut and vaginal microbiota composition Decreased *Lactobacillus* Increased *Proteobacteria* and *Streptococcus* in advanced endometriosis
Hernandes et al. (2020) [24]	Brazil	Case–control	10 vs. 11	Surgical and histopathological confirmation	Vaginal swabs, endometrial and lesion tissue	16S rRNA sequencing (V4 region, Illumina MiSeq)	Reduced *Lactobacillus* in deep endometriosis Increased *Alishewanella*, *Enterococcus*, and *Pseudomonas* species in deep endometriosis
Perrotta et al. (2020) [34]	Brazil/USA	Case–control	35 vs. 24	Laparoscopy and clinical staging (rASRM)	Vaginal and rectal swabs	16S rRNA sequencing (V4 region, Illumina MiSeq)	rASRM stage of endometriosis could be predicted by vaginal microbiota composition *Anaerococcus* abundance and community state type (CST) shifts linked to menstrual phase
Wei et al. (2020) [30]	China	Case–control	36 vs. 14	Histopathological confirmation	Vaginal swabs, endometrial samples	16S rRNA sequencing (V4–V5 regions)	Increased *Prevotella* and *Pseudomonas* Decreased *Lactobacillus*
Shan et al. (2021) [35]	China	Case–control	12 vs. 12	Laparoscopy and histological confirmation	Fecal samples	16S rRNA sequencing (V3–V4 regions, Illumina MiSeq)	Reduced alpha diversity *Firmicutes* increased and *Bacteroidetes* decreased Positive correlations between *Proteobacteria*, IL-17, IL-10, and estradiol levels
Svensson et al. (2021) [21]	Sweden	Cohort	66 vs. 198	Laparoscopy and clinical criteria	Fecal samples	16S rRNA sequencing (V1–V3 regions)	Reduced alpha diversity Altered *Clostridia* and *Bacteroidia* populations
Huang et al. (2021) [23]	China	Case–control	21 vs. 20	Laparoscopy and histology	Fecal samples, cervical swabs, peritoneal fluid	16S rRNA sequencing (Illumina MiSeq)	Increased pathogens in peritoneal fluid Reduced protective taxa in feces *Ruminococcus* and *Pseudomonas* as potential microbial biomarkers in gut and peritoneal fluid, respectively
Le et al. (2021) [36]	USA	Cohort	20 vs. 9	Laparoscopy and pathology	Urine, fecal and vaginal swabs	16S rRNA sequencing (Illumina platform)	Gut microbiota composition associated with oral contraceptive use Higher *Firmicutes*/*Bacteroidetes* ratio
Chang et al. (2022) [37]	Taiwan	Cohort	23 vs. 10	Laparoscopic and histological confirmation	Cervical swabs	16S rRNA amplicon sequencing (Illumina platform)	Reduced cervical microbial diversity correlated with advanced disease stage, higher CA125, and infertility *Lactobacillus jensenii* enrichment and depletion of *Atopobium* and *Prevotella* in severe stages Higher microbial diversity associated with milder symptoms and better clinical outcomes
Pai et al. (2023) [38]	Taiwan	Case–control	37 vs. 35	Histological and imaging diagnosis	Urine and fecal samples	16S rRNA sequencing	Altered gut microbiome composition Reduced diversity and enrichment of *Erysipelotrichaceae*
Hicks et al. (2025) [39]	Australia	Cohort	21 vs. 19	Laparoscopy and histological confirmation	Oral, vaginal, and stool samples	16S rRNA sequencing (V3–V4 regions, Illumina platform)	Distinct microbial signatures across multiple body sites Increased *Fusobacterium* associated with severe disease
Jimenez et al. (2024) [40]	USA	Cohort	35 vs. 15	Laparoscopic confirmation	Rectal and vaginal swabs, cervical lavages	16S rRNA sequencing (Illumina MiSeq)	Increased vaginal *Streptococcus anginosus* and rectal *Ruminococcus* Chronic pelvic pain and endometrioma associated with higher levels of vaginal *Streptococcus*, *Lactobacillus*, and *Prevotella*
Valdés-Bango et al. (2024) [41]	Spain	Cohort	38 vs. 46	Histological and imaging confirmation	Fecal, endometrial, and vaginal samples	16S rRNA sequencing (Illumina platform)	Increased *Ruminococcus gauvreauii* and *Rhodospirillales* in adenomyosis Compositional differences across gut and reproductive tract microbiota.
Do et al. (2024) [42]	USA	Cohort	33 vs. 15	Laparoscopic and histological confirmation	Urine, fecal and vaginal swabs	16S rRNA sequencing (V3–V4 regions)	Altered gastrointestinal microbiota Reduced alpha diversity and distinct beta diversity
Malvezzi et al. (2025) [43]	Brazil	Case–control	27 vs. 23	Laparoscopy and histological confirmation	Peritoneal fluid	16S rRNA sequencing (Illumina MiSeq)	Enrichment of *Flavobacterium*, *Pseudomonas*, and *Bacillus* Peritoneal microbiota triggers inflammasome-related inflammation
Toffoli et al. (2025) [44]	Italy	Cohort	38 vs. 20	Laparoscopy and histological confirmation	Endometrial and vaginal samples	16S rRNA sequencing and ELISA functional assays	Low mannose-binding lectin (MBL) pathway activity associated with dysbiosis Interplay between complement, estrogen pathway genes, and microbial imbalance.
Baușic et al. (2025) [45]	Romania	Cross-sectional	9 vs. NA	Clinical and imaging diagnosis	Fecal samples	16S rRNA sequencing	Significant dysbiosis, with an altered *Firmicutes*/*Bacteroidetes* ratio and elevated *Bacteroidetes* Increased β-glucuronidase and secretory IgA levels as a link between gut microbiota and systemic inflammation

## Data Availability

Data sharing not applicable.

## Supplementary Materials

The following supporting information can be downloaded at: https://www.mdpi.com/article/10.3390/medicina62020351/s1, Table S1: Risk-of-Bias Assessment Using Newcastle–Ottawa Scale (NOS).

## Abbreviations

The following abbreviations are used in this manuscript:

CST	Community State Type
ELISA	Enzyme-Linked Immunosorbent Assay
ERβ	Estrogen Receptor Beta
GnRHa	Gonadotropin-Releasing Hormone Agonist
IL	Interleukin
IVF	In Vitro Fertilization
LPS	Lipopolysaccharide
MBL	Mannose-Binding Lectin
NF-κB	Nuclear Factor kappa-light-chain-enhancer of activated B cells
PRISMA	Preferred Reporting Items for Systematic Reviews and Meta-Analyses
rASRM	Revised American Society for Reproductive Medicine
SCFA	Short-Chain Fatty Acids
TLR4	Toll-Like Receptor 4
TNF-α	Tumor Necrosis Factor-alpha
Treg	Regulatory T Cells
