# Supplementary material for: Gut Microbiota Alterations and Reproductive Tract Dysbiosis in Endometriosis: A Systematic Review
Source: Medicina (Kaunas). 2026 Feb 10;62(2):351. doi: 10.3390/medicina62020351 (PMC12942269; doi:10.3390/medicina62020351)
Supplement: Supplementary file 1 [file medicina-62-00351-s001.zip › medicina-4110119-supplementary.pdf]

Supplementary Table S1. Risk-of-Bias Assessment Using Newcastle–Ottawa Scale (NOS)

Study	Selection (max 4 ★)	Comparability (max 2 ★)	Outcome/Exposure (max 3 ★)	Total Score (max 9 ★)
Khan et al. (2016)	★★★	★	★★	★★★★★★
Wang et al. (2018)	★★★	★	★★	★★★★★★
Ata et al. (2019)	★★★	★★	★★	★★★★★★★
Hernandes et al. (2020)	★★★	★	★★	★★★★★★
Perrotta et al. (2020)	★★★	★	★★	★★★★★★
Wei et al. (2020)	★★★	★	★★	★★★★★★
Shan et al. (2021)	★★★	★	★★	★★★★★★
Svensson et al. (2021)	★★★	★★	★★	★★★★★★★
Huang et al. (2021)	★★★	★	★★	★★★★★★
Le et al. (2021)	★★★	★★	★★	★★★★★★★
Chang et al. (2022)	★★★	★★	★★	★★★★★★★
Pai et al. (2023)	★★★	★	★★	★★★★★★
Hicks et al. (2024)	★★★	★★	★★	★★★★★★★
Jimenez et al. (2024)	★★★	★★	★★	★★★★★★★
Valdés-Bango et al. (2024)	★★★	★★	★★	★★★★★★★
Do et al. (2024)	★★★	★★	★★	★★★★★★★
Malvezzi et al. (2024)	★★★	★	★★	★★★★★★
Toffoli et al. (2025)	★★★	★★	★★	★★★★★★★
Bausic et al. (2025)	★★	★	★	★★★★
